# Women's knowledge and attitudes toward female genital mutilation and associated factors in Diguna Fango, a rural district in southern Ethiopia: a community-based mixed study

**DOI:** 10.3389/fgwh.2025.1516925

**Published:** 2025-04-15

**Authors:** Tamirat Beyene Gerete, Asresash Demissie, Enatfenta Sewmehone, Wubishet Gezimu

**Affiliations:** ^1^ School of Midwifery, College of Health Sciences and Medicine, Wolaita Sodo University, Wolaita Sodo, Ethiopia; ^2^ School of Nursing, Institute of Health, Jimma University, Jimma, Ethiopia; ^3^ Department of Nursing, College of Health Sciences, Mattu University, Mattu, Ethiopia; ^4^ Department of Health Behaviour and Society, Institute of Health, Jimma University, Jimma, Ethiopia

**Keywords:** knowledge, attitude, female genital mutilation, diguna fango, Ethiopia

## Abstract

**Background:**

Female genital mutilation (FGM) is a widely practiced custom in Ethiopia. The women's knowledge and attitudes toward it and influencing factors have not been explored in Ethiopia, particularly in the rural districts. Hence, this study assessed women's knowledge and attitudes toward female genital mutilation and associated factors in Diguna Fango, a rural district in southern Ethiopia.

**Methods:**

This study adopted a community-based cross-sectional study design using a sequential mixed-method explanatory approach. It was conducted from May 20, 2023, to June 30, 2023, among 821 participants selected using a multistage sampling technique. The quantitative data were collected using a structured interviewer-administered questionnaire. A key informant interview was conducted to collect the qualitative data. A binary logistic regression analysis was conducted to identify factors associated with the outcome variables. A *p*-value <0.05 at a 95% CI was used to declare statistical significance.

**Results:**

Of the 821 participants included in the study, 53.2% had good knowledge and 46% had an unfavorable attitudes towards female genital mutilation, respectively. Monthly income (AOR = 1.61; 95% CI: 1.39–2.95) and partners’ educational status (AOR = 2.17; 95% CI: 1.37–4.89) were significantly associated with knowledge, whereas being a government employee (AOR = 2.12; 95% CI = 1.45–3.11) and private employee (AOR = 3.99; 95% CI = 1.63–6.77), having student partners (AOR = 2.64; 95% CI = 1.40–4.95), circumcision history (AOR = 2.58; 95% CI = 1.41–4.71), and knowledge (AOR = 1.48; 95% CI = 1.11–1.98) were shown to be associated with attitude towards female genital mutilation. Moreover, sociocultural drivers, awareness of adverse health effects, religious attributes, and sexuality concerns were explored as attributes of knowledge and attitudes toward female genital mutilation/cutting.

**Conclusion:**

Compared to previous similar local and global findings, lower levels of knowledge and higher levels of support for female genital mutilation were observed in the area. Sociocultural, religious, and sexual concerns influence knowledge and attitudes toward female genital mutilation. Therefore, the concerned bodies need to mobilize the community and work closely with the health development armies and religious institutions to boost women's knowledge and change favorable attitudes towards FGM/C.

## Introduction

Female genital mutilation or cutting (FGM/C), a subset of harmful traditional practices (HTP), is defined as partial or complete removal of the genitalia for nontherapeutic reasons (1). The FGM procedures may be performed in different ways, including clitoridectomy (partial or complete removal of the clitoris), excision (the removal of the clitoris and labia minora), infibulation (covering or sealing the vaginal orifice), and others like genital pricking, piercing, incising, scraping, and cauterization (2).

FGM/C can cause short-term and long-term health effects on the victim. Pain, bleeding, and infections are some of the short-term complications, whereas genital lacerations (fistula), infertility, and reduced sexual desire are the long-term complications of FGM/C. Both of these complications can put the victim at risk of death and disability (3, 4). In addition to its adverse effects on the victim's health, it can increase health costs (5). Moreover, FGM/C violates girls’ or women's human rights by impeding their physical and mental integrity (6).

Despite its adverse effects, FGM/C is practiced in almost all nations and mankind in the world. Globally, around 200 million girls and women undergone FGM/C (7). It is a common and long-practiced custom in Africa, Asia, and the Middle East. It is also practiced in America, Europe, and the United Kingdom (8). In Ethiopia, about 65% of reproductive-age women practice FGM/C. The highest prevalence was observed in the Somali region, and the lowest was in Tigray. Almost all ethnic groups in Ethiopia practice FGM/C. However, it is variably distributed and more regarded among some ethnic groups. For instance, it is highly practiced in the Afar (98%) and Somali (99%) women, then followed by Hadiya and Wolaita (each accounted for 92%) (9, 10).

The FGM/C practice is related to some sociocultural and religious beliefs (11). According to some traditions, uncircumcised girls or women are excluded from marriage and social support networks (12). For instance, a demographic and health survey from the United States of America (USA) revealed that FGM/C was practiced for social acceptance, marriageability, community belonging, proof of virginity, reducing promiscuity, hygiene, and religion (13). A study conducted in the Somali community in Kenya found that culture and religion were important influencers of the community's perception of FGM/C. According to the same study, the community believed that a girl's sexual purity could be conserved by compromising her sexual desires until marriage (14).

It is believed that identifying people's attitudes and knowledge and their influencing factors is the primary step in ending FGM/C (13, 15–17). The World Health Organization (WHO) identified knowledge gaps as a major obstacle in the elimination programs of FGM/C (18). However, evidence shows that there is poor knowledge of FGM/C and an unfavorable women's attitude in both local and global contexts. For instance, a study from the Iraqi Kurdistan region found little knowledge of FGM/C (19). According to a study from Ekiti, Nigeria, 47% of women did not know about FGM/C (20). A study from the Somali community in Kenya revealed that only 14.6% of participants were not knowledgeable about FGM/C (14). In Ethiopia, the highest knowledge of FGM/C was observed in the Amhara region (21), and the lowest was found in the Somali region (22). Various sociodemographic factors influence women's knowledge and attitude toward FGM/C. These factors include age, residence, level of education, religion, marital status, and income (21, 23).

A finding from Nigeria revealed that 70% of respondents had unfavorable (good) attitudes toward FGM/C (20). Another cross-sectional study from Khartoum State found that 25.5% of participants had unfavorable attitudes toward FGM/C (24). A study from the Degadamot district of the Amhara region of Ethiopia found that about 54.2% of participants had favorable attitudes toward FGM (21). Various studies from Ethiopia, including Jigjiga district, Hababo Guduru district, and Angacha Woreda of Kembata Zone, found that about 62.7%, 53%, and 49.6% of participants had unfavorable attitudes toward FGM/C, respectively (25–27).

Girls’ or women's attitudes toward FGM/C are also attributed to some socio-demographic and individual factors. A survey conducted in 12 sub-Saharan African countries identified age, educational status, marital status, place of delivery, frequency of antenatal care, and a history of circumcision as factors affecting women's attitudes toward FGM/C. Similarly, a cross-sectional study from Egypt determined that rural residence, previous circumcision history, and poor knowledge influence attitudes toward FGM/C (28). Another Egyptian nationwide study identified that educational status, marital status, previous circumcision history, and knowledge affect an individual's attitude toward FGM/C (29). Moreover, the educational status of an individual was found to be a factor affecting their attitude toward FGM/C (30).

The Ethiopian government promised to increase the national budget allocation by 10% to end FGM/C by 2025. This is in line with the 2014 Girl's Summit in London, which aimed to end childhood marriage and FGM/C. However, the national prevalence of FGM/C practice has decreased only by 0.8% annually (31). Particularly, the current FGM/C practice is significantly higher in the study area. For instance, a study conducted in 2021 found that 88.9% of FGM/C practices were in the Boditi district of the Wolaita Zone (32). This study hypothesized that the persistence of the FGM/C practice despite different interventions implemented might be related to knowledge and attitude gaps and some socio-cultural and religious influences. Based on this hypothesis, the current study assessed the women's knowledge and attitudes toward FGM/C. It explored the sociocultural and religious attributes that affect FGM/C in the Diguna Fango district of southern Ethiopia. The findings of this study will benefit the concerned bodies in setting up community-based intervention strategies, considering newly identified influencers.

## Methods and materials

### Area and period

The study was conducted in the Diguna Fango district from May 20 to June 30, 2023. The Diguna Fango district is one of the 12 rural districts in the Woliata Zone of southern Ethiopia. It is located 375 km southwest of Addis Ababa, the capital of Ethiopia. The district is administratively divided into 32 Kebeles (Ethiopia's lower administrative units). According to the 2007 Central Statistics Agency (CSA) report, 24,702 households and 121,040 people inhabited the area. During the study period, a total of 37 (one primary hospital, 5 health centers, and 31 health posts) public health institutions were actively serving the catmint population in the district.

### Study design

This study adopted a community-based cross-sectional study design using a sequential mixed-method explanatory approach.

### Study participants and eligibility criteria

For the quantitative study, all reproductive-age women (15–49) of the Diguna Fango district were considered the source population, whereas all sampled women who were recognized as permanent residents (who lived for at least 6 months) of the selected Kebeles were included in this study. However, women who were severely sick during the data collection were excluded from the study.

Key informants (KIs) thought to have a deeper understanding of FGM/C were purposefully selected from different governmental offices, religious organizations, community leaders, and local elders of the qualitative data.

### Sample size calculation and sampling technique

The sample size for the first objective was estimated using a single population proportion formula, $n=[Z(\alpha /2){]}^{2}\;P(1-P)/d^{2}$. A 95% confidence level, a 5% degree of precision, a population proportion of knowledge (55.4%), and unfavorable (good) attitudes toward FGM/C (49.6%) (27) were assumed during the estimation. For the second objective, a double population proportion formula was used to calculate sample size using the EPI-INFO version 7.25.0 computer software, assuming a power of 80%, a 95% confidence level, and a 1:1 exposed to unexposed ratio. However, the sample size calculated for the knowledge outcome of *n* = 384 was found to be the highest, and the final sample size of *n* = 845 was utilized after multiplying by 2 design effects and adding 10% non-response rates.

**Figure 1:**
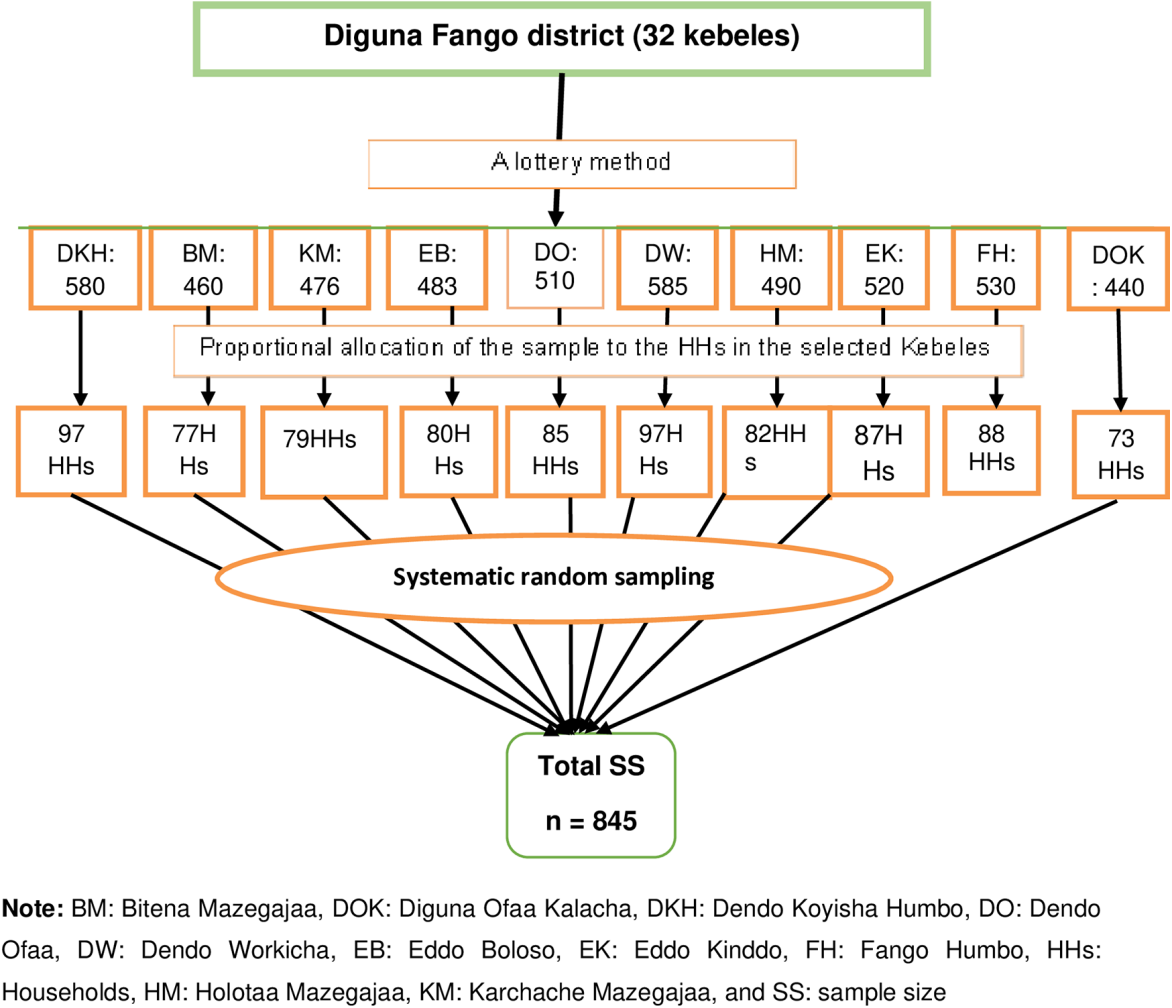
A diagrammatic presentation of the sampling techniques used to assess women's knowledge and attitudes toward female genital mutilation in Digun Fango, a rural district in Southern Ethiopia: A community-based mixed study.

The participants were selected using a multistage sampling technique. Of the thirty-two Kebeles in the district, ten (30%) were selected using a lottery method. In the second sampling stage, a systematic random sampling technique was used to select each participant (Figure 1). The *K*^th^ value of 6 was obtained by dividing 5,074 (the estimated total of households with women of the reproductive age group (15–49) in the selected Kebeles) by 845 (the sample size). A lottery method was used to select the first house, and then every sixth house was visited. In cases where more than one reproductive-age woman was present in the selected households, a lottery method was used to select a participant.

For the qualitative segment, 11 key informants (two Kebele women representatives, a district court officer, a district health officer or healthcare provider, a district women and children affairs officer, two local elders, two religious leaders, a traditional circumciser, and a health extension worker) were purposefully selected and interviewed based on the information saturation.

### Study variables

Knowledge of FGM/C and attitudes toward it were the outcome variables of this study. The socio-demographic characteristics (age, number of daughters, marital status, residence, ethnicity, educational status, religion, occupation status, and monthly income) and parental factors (decision-making power, maternal health service, history of FGM/C, family support for the continuation of FGM/C, reason for family support for the continuation of FGM/C, and the way they think to stop FGM/C) were tested to identify their independent association with each other's knowledge and attitudes toward FGM/C.

### Operational definitions

FGM/C is defined as all procedures involving partial or total removal of the external female genitalia or other injuries to the female genital organs, whether for cultural or other non-therapeutic reasons (1).

#### Knowledge

It was assessed by ten FGM/C knowledge-related questions with “yes” or “no” responses. A score of “1” was given for every correct response and “0” for every incorrect response. Then the sum of each item in individual scores was converted to a percentage. Finally, the total percentage score of 50.0% and above was categorized as good knowledge of FGM/C, whereas less than 50.0% was categorized as poor knowledge of FGM/C from ten questions about FGM/C (26).

#### Attitude

Participants’ attitude toward FGM/C was assessed using ten questions. Each question contains a five-point Likert scale assigned as strongly disagree = 1, disagree = 2, neutral = 3, agree = 4, and strongly agree = 5. Participants who scored greater than or equal to the median value of 10 attitude-measuring questions were categorized as having a favorable (FGM/C-supporting) attitudes toward FGM/C). In contrast, participants who scored less than the median value were categorized as having an unfavorable (good) attitudes FGM/C) (21).

### Data collection tool and techniques

An interviewer-administered tool was used for the data collection. The data collection tool was adapted from the 2016 EDHS (10) and reviewed from similar studies (6, 20–24, 27, 33, 37). The tool consists of questions about socio-demographic characteristics, knowledge, and attitudes towards FGM. The English version tool [Supplementary S1] was translated to the Wolaytatto doona (local language) [Supplementary S2] and back to English by language experts who are fluent in both languages. Four graduate nurses and two health professionals with postgraduate degrees collected the data and supervised the data collection process, respectively.

The qualitative data were collected according to Tong et al.'s consolidated criteria for reporting qualitative research (COREQ) guideline (34) [Supplementary S3]. Two graduate professionals (a male midwife and a nurse) conducted a key informant interview (KII) using a key informant guide that contained a central question and associated sub-questions. The two interviewers speak and understand the language and values of the interviewees. However, the interviewers had no prior relationship with the participants, and the interviewees did not know about the personal goals of the interviewers. The interviewers contacted the interviewees a week before the interview to adjust a convenient time and place. A maximum of 30 min of face-to-face interviews were conducted in the interviewees’ preferred (private and quiet) locations, including their homes, offices, and fields. There was no incentive or benefit agreement made between the interviewers and interviewees. Sound recordings (using mobile phone) and field notes were taken during the interview sessions.

### Data quality control and management

Two days of training were given to the data collectors and supervisors regarding the tool's content and participants’ rights. A pre-test was conducted among 42 participants, 5% of the total sample size, in the Dimttu kebele, one of the non-selected kebeles in the Diguna Fango districts. Then, necessary amendments were made to the instructions, contents, and the sequence of questions based on the results. The investigators and supervisors checked the data collection process for completeness, accuracy, clarity, and consistency on a daily basis. The data were double-entered and cleaned before analysis. To secure the quality of qualitative data, the audio records were attentively listened to get insight, and the field notes were rechecked and re-read extensively.

### Data processing and analysis

The collected data were coded and entered into Epi Data 3.1 version statistical software and then exported to Statistical Product and Service Solutions (SPSS) version 26 software for analysis. The descriptive statistics were presented using text narration, frequency tables, proportions, and charts. A binary logistic regression model was used to test the association between dependent and independent variables. All the logistic regression model assumptions, including multicollinearity, were checked using the variance inflation factor (VIF). All the eligible variables in the bivariate analysis (a *p*-value <0.25) were further analyzed in the multivariable model. The Hosmer and Leme-Shew goodness-of-fit tests were used to assess the model fitness. An adjusted odds ratio (AOR) was used to show the strength and direction of the association, whereas a *p*-value of <0.05 at a 95% confidence interval (CI) was used to assert statistical significance.

Audio records in Wolaytatto Doona were transcribed and translated into English. Two authors coded the data segment by segment using open codes with the support of Atlas Ti version 7.5 after reading and re-reading the transcript extensively. Accordingly, four themes emerged during the coding process. Then, the themes were triangulated with the quantitative findings for discussion.

### Ethical approval and informed consent

This study followed the ethical principles (35). The ethical clearance letter (Ref. No.: JUIH/IRB/385/23) was obtained from the Jimma University Institute of Health (JUIH) Institutional Review Board (IRB). The study was conducted after getting permission from the district administrative officers. Then, each participant was informed about the study and signed a written informed consent form. The participants who were unable to read and sign gave verbal consent. Assent was obtained from those non-autonomous (<18 years old) participants. The participants’ guardians signed the consent. The study tool used anonymity to keep the participants’ information confidential. The audio files were kept with the principal investigator and locked with passwords to prevent the spread of the interviewees’ voices.

## Results

### Socio-demographic characteristics of the respondents

A total of 821 women participated in the quantitative study. About one-third (33%) of the participants were in the 25–29 age range, with a mean age of 27.6 ± 5.4 years. The majority, 694 (84.5%), of the participants were married. Four hundred eighty-one (58.6%) and 523 (63.7%) of participants and their partners attended secondary education, respectively. Four hundred seventy-four (57.7%) participants were housewives (Table 1).

**Table 1:** Socio-demographic characteristics women in Diguns Fango, a rural district in Southern Ethiopia: A community-based cross-sectional study using mixed method, 2023.

Variables	Categories	Frequencies (*n* = 821)	Percent (%)
Age	15–19	41	5
	20–24	191	23.3
	25–29	271	33
	30–34	203	24.7
	35–39	99	12.1
	40–44	16	1.9
Religious	Orthodox	144	17.5
	Protestant	555	67.6
	Muslim	11	1.3
	Catholic	76	9.3
	Others^a^	35	4.3
Ethnicity	Welayta	765	93.2
	Dawuro	21	2.6
	Gamo	7	0.9
	Others^b^	28	3.4
Educational status	Not attended formal education	105	12.8
	Attended primary (1–8 grades) education	164	20.0
	Attended secondary (9–12 grades) education	481	58.6
	Attended college and above	71	8.6
Occupation	House wife	474	57.7
	Private worker	26	3.2
	Governmental employee	156	19.0
	Merchant	145	17.7
	Student	20	2.4
Marital status	Single	4	0.5
	Married	694	84.5
	Divorced	89	10.8
	Widowed	30	3.7
	Separated	4	0.5
Partner education status	No formal education	15	1.8
	Primary	174	21.2
	Secondary	523	63.7
	Above secondary	109	13.3
Partner's occupation	Farmer	355	43.2
	Governmental/NGO employment	124	15.1
	Merchant	238	29.0
	Student	51	6.2
	Daily workers	53	6.5
Family monthly income	<500	220	26.8
	500–999	224	27.3
	1,000–1,499	68	8.3
	1,500–1,990	77	9.4
	≥2,000	232	28.3

^a^ Cultural believers and pagans.

^b^ Oromo, Amhara, Hadiya, Kambata, Silte, and Gurage.

### Key informants’ profile and themes developed

In the qualitative part of this study, a total of eleven interviewees participated. Of these, two were 32- and 34-year-old married women whose roles were women's chairpersons in the community; two were 38- and 40-year-old married religious leaders; a 45-year-old married uneducated female circumciser; a 34-year-old married a degree-holder male who was being worked as a district women and child affairs (WCA); two (49- and 51-year-old females who attended primary education and not attended formal education, respectively) were local leaders; a 36-year-old married diploma-holder lawyer; and two (34-year-old married a BSc-holder male and a 33-year-old married female diploma-holder) were a district health officer and a health extension worker, respectively.

Four main themes emerged during qualitative data coding and analysis, including awareness and understanding of the impacts of FGM/C, socio-cultural attributes of FGM/C, religion as an influencer of FGM/C, and sexuality as an influencer of FGM/C. These themes are narrated and quoted by mentioning the interviewees’ KII and types.

### Participants’ knowledge of female genital mutilation/cutting

**Figure 2:**
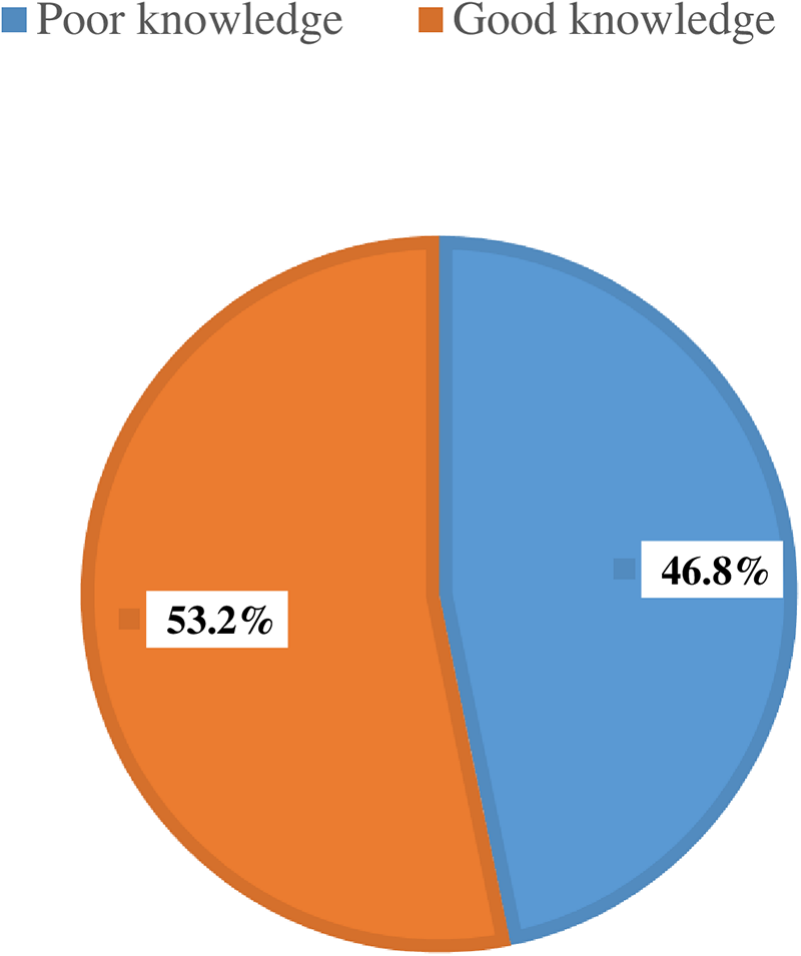
Women's knowledge of female genital mutilation/cutting in Digun Fango, a rural district in southern Ethiopia: A community-based mixed study, 2023 (*n* = 821).

Generally, more than half (437 (53.2%); 95% CI: 49.8%, 56.5%) of the participants had good knowledge of FGM/C practice (Figure 2).

Table 2 shows the participants’ responses to knowledge-measuring questions. Around 567 (69.1%) participants stated that FGM/C has no health benefits. Nearly three-fourths (70.9%) of the participants knew that FGM/C is an HTP, and around 522 (63.7%) mentioned that FGM/C is an illegal practice. However, nearly half (47.4%) of them did not know about FGM/C-related complications. This result was supported by a qualitative finding from KII that most participants undermined the complications of FGM/C and considered the procedure to have no problem. For instance, a KI stated:

**Table 2:** Women's knowledge of female genital mutilation/cutting in Diguna Fango, a rural district in Southern Ethiopia: A community-based cross-sectional study using mixed method, 2023 (*n* = 821).

Knowledge questions	Yes (%)	No (%)
I have information about female genital circumcision	821 (100)	0
I know FGM has health complication	432 (52.6)	389 (47.4)
I can classify it as immediate or long term complication	100 (12.2)	721 (87.8)
I know FGM can decrease sexual pleasure	322 (39.2)	499 (60.8)
I know FGM is harmful traditional practices	582 (70.9)	239 (29.1)
I know FGM brings complication during delivery	347 (42.3)	474 (57.7)
I know the FGM has no health benefits	567 (69.1)	254 (30.1)
I know FGM is forbidden in the law	522 (63.7)	299 (35.3)
I know FGM is a violation of the rights of the girl-child	332 (40.4)	489 (59.6)
I know the different forms of FGM	67 (8.2)	754 (91.8)

“…Regarding complications, after the genital cut is done, the grandmother and other elders provide favor to the girl by saying ‘**Kana-Kafo masuntta gido’** (a proverb in the local language), meaning may it heal soon. And also, we put butter on the wound to facilitate healing within short periods (3 or 4 days) unless it has no problem…” (KI5)

### Participants’ awareness and understanding of impacts of FGM/C

The female KIs openly discussed their FGM/C experiences and those of their daughters, whereas the male KIs shared stories about their daughters and sisters or stories they had heard about short- and long-term adverse health effects. The majority of female KIs agreed that women are generally more supportive of FGM/C than men because they believe the procedure is harmless and has no negative health consequences. However, a few KIs rejected the mentioned idea. For instance, a KI stated that it has physical health problems but is considered minor.

“…it [FGM] has long-term complications like increasing chances of prolonged labor and short-term complications like pain; however, the girls relieve easily due to the cultural celebrations. But most of our communities do not accept that it has a problem, and they think it is cutting only the sensitive parts with no harm at all, or they consider the complications minor…” (KI1).

Another KI also mentioned that people in his community do not know the complications of FGM/C. He stated:

“…we do not know its (FGM/C) advantages. We do not understand its complications. Uneducated elderly women do it in an unsterilized field…” (KI1).

### Participants’ attitude toward female genital mutilation/cutting

**Figure 3:**
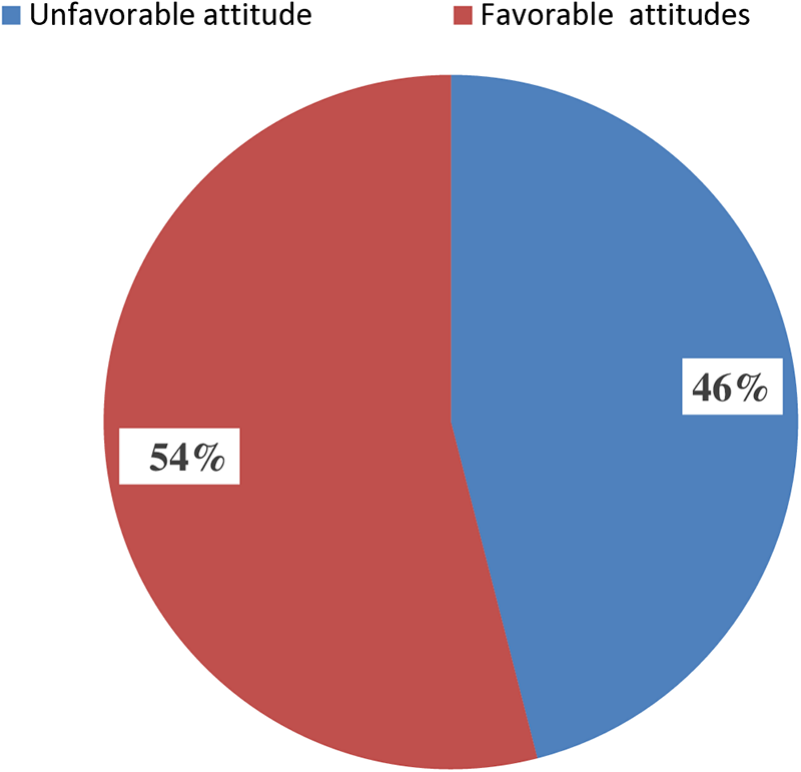
Women's attitudes toward female genital mutilation/cutting in Digun Fango, a rural district in southern Ethiopia: A community-based mixed study, 2023 (*n* = 821).

The study found that less than half (46%; 95% CI: 42.6%, 49.3%) of the participants had unfavorable attitudes toward FGM/C (Figure 3).

Table 3 shows the participants’ responses to attitude-related questions. More than half (55.2%) of the participants did not decide to circumcise their daughters voluntarily.

**Table 3:** Women's attitudes towards female genital mutilation/cutting in Diguna Fango districts of southern Ethiopia: A community-based cross-sectional study using mixed method, 2023 (*n* = 821).

Attitude question	Strongly disagree	Disagree	Neutral	Agree	Strongly agree
I support FGM	97 (11.8%)	200 (24.4%)	324 (39.5%)	182 (22.2%)	18 (2.2%)
FGM can protect virginity of female	22 (2.7%)	183 (22.3%)	354 (43.1%)	214 (26.1%)	48 (5.8%)
I think uncircumcised females are not faithful for marriages	19 (2.3%)	127 (15.5%)	373 (45.4%)	247 (30.1%)	55 (6.7%)
I think FGM practice is required by my religion	45 (5.5%)	210 (25.6%)	467 (56.9%)	91 (11.1%)	8 (1.0%)
I think uncircumcised females have increased sexual feeling	18 (2.2%)	223 (27.2%)	314 (38.2%)	218 (26.6%)	48 (5.8%)
I think FGM is good practice	40 (4.9%)	316 (38.5%)	376 (45.8%)	79 (9.6%)	10 (1.2%)
I think circumcised female has no problem during child birth	64 (7.8%)	141 (17.2%)	197 (24.0%)	373 (45.4%)	46 (5.6%)
I voluntarily circumcise if I have a daughter	25 (3.0%)	122 (14.9%)	453 (55.2%)	198 (24.1%)	23 (2.8%)
I think uncircumcised female is called as a maid in societies	18 (2.2%)	108 (13.2%)	373 (45.4%)	237 (28.9%)	85 (10.4%)
I agree with FGM continuity for the future	18 (2.2%)	107 (13.0%)	479 (58.3%)	206 (25.1%)	11 (1.3%)

About 58.3% of the participants agreed to continue the FGM/C practices in the future. Furthermore, this finding was supported by the qualitative finding. For instance, a KI stated:

“…This practice really will continue in the future because of what our parents have instructed us to do. Even if we want to stop it, it may take time to stop…” (KI4).

### Sexuality as an influencer of FGM/C

About 45.4% of the participants thought that uncircumcised females were unfaithful in their marriages (Table 3). The qualitative finding also supported this result. Accordingly, the participants mentioned that FGM/C is primarily practiced to compromise female sexual desires, to avoid premarital sexual initiation and relations, to make a woman faithful to her marriage, and to have a single partner before and after marriage. For instance, a KI stated:

“…Female circumcision is important to reduce the power of sexuality. An uncircumcised woman is hypersexual and dirty. So, she might consume more energy from her husband unless she needs an additional sexual partner to have frequent sexual intercourse. If the husband is at work or far from home, the wife might not be able to control her sexual desire…” (KI2).

A KI added:

“Regarding sexual feeling, they enjoy sex much more than circumcised women.” Female circumcision is crucial to reducing the power of sexuality often requested by a second partner other than a husband…” (KI11).

Another KI stated:

“…From my point of view, an uncircumcised girl is unfaithful to her husband, and such a girl is filthy and hypersexual. An uncircumcised wife might not be able to control her sexual desire when her husband is at work or away from home…” (K I4).

Moreover, it is thought that FGM/C could protect a woman from a premarital sexual relationship and maintain her virginity. A KI supported this statement as follows:

“…There are reasons to carry out female circumcision, such as to prevent premarital sex because an uncircumcised girl has a higher emotion that initiates them to start sexual intercourse, in case the girl has sensitive parts…” (KI2).

### Religion as an influencer of FGM

Four hundred sixty-seven (69%) participants agreed that their religion permits the FGM/C practice. The qualitative study also explored the women's tendency to uphold the FGM/C practice in the community as a result of their religious convictions. They believe that FGM/C is carried out to make a girl religiously acceptable and to keep her clean or prepared for religious instruction.

A KI supported the above statements by stating:

“…Additionally, circumcised girls, according to what my grandparents told me, respect other people and are modest. We held the opinion that female circumcision was acclaimed by religion as a condition for baptism and serving in the church…” (KI5)

Another KI added:

“…Female circumcision is widely practiced because of societal perspectives such as having a fear of God or respecting one's grandparents’ cultural or religious beliefs. Although I don't know the passage or verses in the Bible, the girl will not serve in the church unless she gets circumcised…” (KI11).

However, a KI stated that there is no religious ground to do FGM/C; rather, the practice is based on ideas of tradition and culture that their parents had instilled in them. He stated:

“…Considering that it is not a requirement of religion, it must be stopped. We are far from that tradition; it is a matter of culture. Neither the Old nor the New Testaments of the Holy Bible support female circumcision. I oppose it. It doesn't say to remove certain tissues from girls. Thus, it should be considered a wrong practice because God created women in good health. However, our community views it as an example of our religious fundamental teachings and heroes in cultural practices…” (KI6)

### Parental related variables of respondents

The majority, 768 (93.5%), of the participants had been circumcised. About 386 (47.0%) of the FGM/C decisions were made by the respondents’ mothers. This finding is in line with the qualitative finding. For instance, a KI said that:

“… Mothers always set plans to circumcise their daughters early because, when growing up, it may be painful, and the girl may have increased sexual desire, which makes her shy to go outside the home out of confidence. Also, we ask for fathers’ decisions to circumcise our daughters…” (KI4).

**Figure 4:**
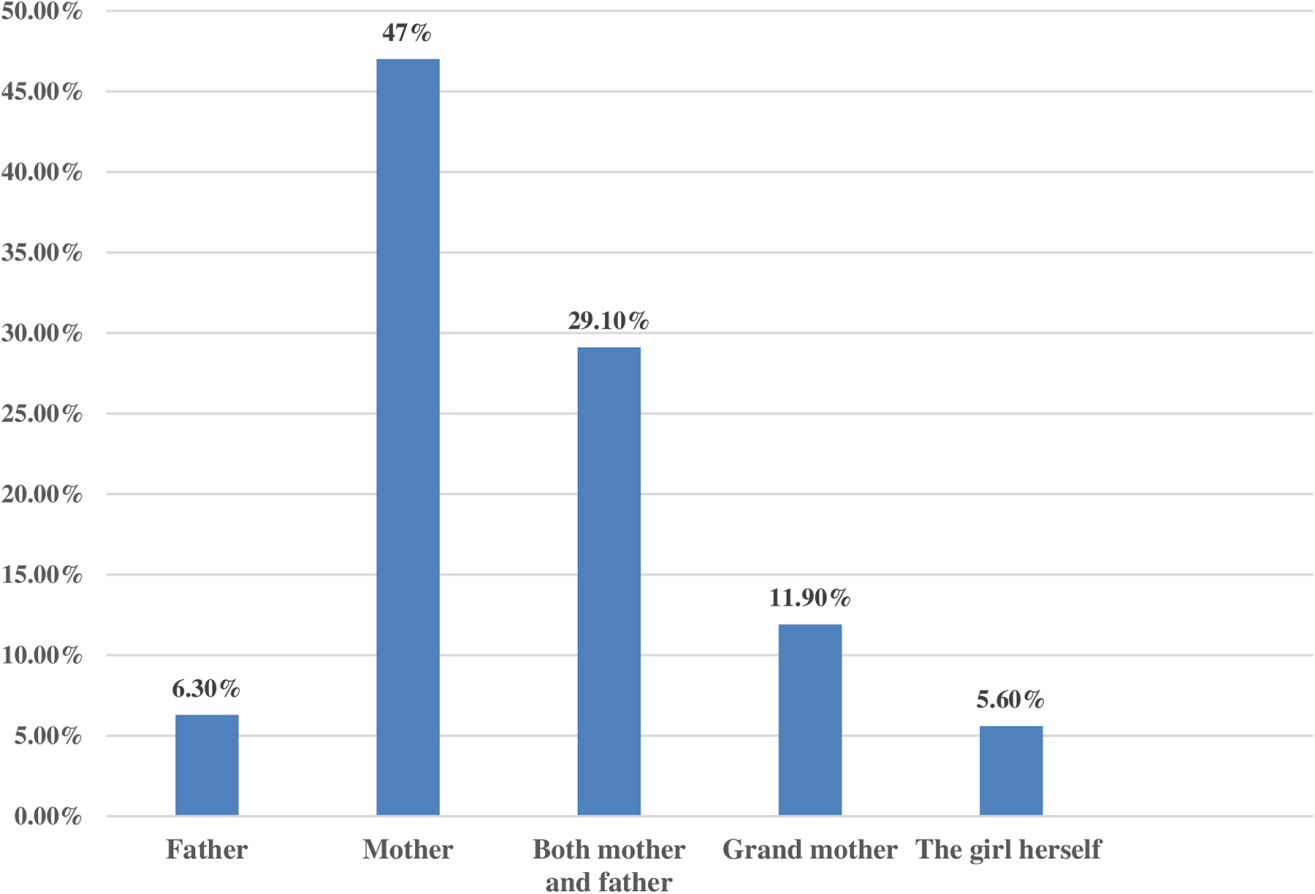
Decision-makers to perform female genital mutilation among family members in Digun Fango, a rural district in Southern Ethiopia: A community-based mixed study, 2023.

About 705 (85.9%) of the participants’ families supported the continuation of the FGM/C. More than half, 416 (50.7%), of the participants stated that enforcing legislation is the best way to stop FGM/C (Figure 4 and Table 4).

**Table 4:** Parental factors related variables of respondents about FGM/C practice in Diguna Fango, a rural district in southern Ethiopia: A community-based mixed study, 2023 (*n* = 821).

Variables		Frequency (*n* = 821)	Percent
Your family member who decides to perform FGM	Father	52	6.3
	Mother	386	47.0
	Both mother and father	239	29.1
	Grand mother	98	11.9
	The girl herself	45	5.5
	Relatives	1	0.1
I have history of ANC visit/place of delivery at health institution	Yes	765	93.2
	No	56	6.8
I get circumcised	Yes	768	93.5
	No	53	6.5
My family support the continuation of the FGM	Yes	705	85.9
	No	116	14.1
I Know the reason of my family support of the continuation of the FGM	Yes	729	88.8
	No	91	11.1
	I do not know	1	0.1
The best way to stop female circumcision	Enforced legislation	416	50.7
	Educational campaign to women	115	14.0
	Improvement of status of women	179	21.8
	Fathers should take more responsibility	95	11.6
	Sexual education	14	1.7
	Other religious leader	5	0.6

This finding is also in line with the qualitative finding. For instance, a KI said that:

“….So, in my opinion, to stop this practice, we have to work more with local leaders and health extension workers at Kebele to get information, educate girls at schools and mothers at home, and enforce legislation on mothers who circumcise their daughters…” (KI2).

### Factors associated with participants’ knowledge of female genital mutilation

Table 5 shows the bivariate and multivariable logistic regression analysis results of factors associated with participants’ knowledge of FGM/C. Six variables, including maternal religion (*P* = 0.223), maternal education (*P* = 0.008), maternal occupation (*P* = 0.004), monthly income (*P* = 0.000), partners’ occupation (*P* = 0.022), and partners’ education (*P* = 0.077), showed an association with the knowledge of FGM/C in the bivariable analysis. However, maternal education, partner's education, and monthly income were shown to have independent associations in the multivariable analysis.

**Table 5:** Factors associated with women's knowledge of FGM/C in Diguna Fango, a rural district in southern Ethiopia: A community-based mixed study; 2023 (*n* = 821).

Variable	Categories	Knowledge of FGM/C		Odd ratio with 95% CI		*P*-value
		Good	Poor	COR	AOR	
Monthly income (ETB)	<500	134 (60.9%)	86 (39.1%)	1	1	
	500–999	92 (41.1%)	132 (58.9%)	0.45 (0.23–1.15)	1.61 (1.39–2.95)	0.031*
	1,000–1,499	32 (47.1%)	36 (52.9%)	0.57 (0.23–0.89)	1. 02 (0.75–1.34)	0.487
	1,500–1,999	41 (53.3%)	36 (46.7%)	0.73 (0.25–1.01)	0.99 (0.76–1.15)	0.792
	≥2,000	138 (59.5%)	94 (40.5%)	0.94 (0.66–1.21)	0.74 (0.25–1.01)	0.777
Partners’ educational status	Not attended formal education	6 (40.0%)	9 (60.0%)	1	1	
	Attended primary education	111 (63.8%)	63 (36.2%)	2.64 (0.999–3.17)	0.72 (0.49–1.14)	0.473
	Attended secondary education	251 (48.0%)	272 (52.0%)	1.38 (0.92–2.68)	0.79 (0.42–1.26)	0.885
	Attended college and above	69 (63.3%)	40 (36.7%)	2.58 (1.35–5.82)	2.17 (1.37–4.89)	0.024*

* Statistically significant variables.

1, reference category; ETB, Ethiopian birr.

In this study, participants whose partners studied college and above were about 2 times more likely to have good knowledge of FGM/C than those whose partners did not attend formal education (AOR = 2.17; 95% CI: 1.37–4.89). Themes from the qualitative finding also supported this association. For instance, a KI said that:

“… No one has doubted why it is done and objected to the practice. Uneducated partners always make fun of circumcised mothers. Every member accepts it as the norm. But nowadays, in our society, some educated mothers understand its benefits and risks, and they are saying it is harmful and not necessary…” (KI1).

Uniquely to the previously mentioned ideas, a KI shared the following idea:

“Woman's circumcision has the potential to cause issues for the wife and her husband. The mothers who are educated stress that FGM/C is a terrible experience that has psychological effects, despite the stigma and fear that have existed in the community up until this point…” (KI3).

Moreover, participants who earn a monthly income of 500–999 Ethiopian birrs (ETB) were about 61% more likely to have good knowledge of FGM/C compared to participants whose income was less than 500 ETB (AOR = 1.61; 95% CI: 1.39–2.95).

### Factors associated with participants’ attitudes toward female genital mutilation/cutting

Table 6 shows the bivariable and multivariable logistic regression analysis results of factors associated with participants’ attitudes toward FGM/C. Of the eleven variables, including maternal education (*P* = 0.001), maternal occupation (*P* = 0.000), monthly income (*P* = 0.014), partners’ occupation (*P* = 0.002), partners’ education (*P* = 0.067), maternal knowledge of FGM/C (*P* = 0.002), history of circumcision (*P* = 0.003), family members’ decision to perform FGM/C (*P* = 0.144), ethnicity (*P* = 0.116), frequency of ANC visit or place of delivery at a health institution (*P* = 0.243), and the way they thought to stop FGM/C (*P* = 0.153), were shown to be associated with the participants’ attitude toward FGM/C in the bivariable analysis.

**Table 6:** Factors associated with women's attitudes toward FGM/C in Diguna Fango district of southern Ethiopia: A community-based mixed study; 2023 (*n* = 821).

Variable	Categories	Attitudes toward FGM/C		Odd ratio with 95% CI		*P*-value
		Unfavorable	Favorable	COR	AOR	
Maternal occupation	House wife	280 (59.1%)	194 (40.9%)	1	1	
	Private	7 (27.0%)	19 (73.0%)	0.25 (0.10–0.62)	3.99 (1.63–6.77)	0.002*
	Governmental employment	60 (38.5%)	96 (61.5%)	0.43 (0.30–0.63)	2.12 (1.45–3.11)	0.001*
	Merchant	85 (58.6%)	60 (41.4%)	0.98 (0.67–1.43)	0.96 (0.66–1.45)	0.900
	Student	11 (55.0%)	9 (45.0%)	0.85 (0.34–2.08)	0.94 (0.37–2.37)	0.888
Partners’ Occupation	Farmer	209 (58.9%)	146 (41.1%)	1	1	
	Governmental employee	66 (53.2%)	58 (46.8%)	0.80 (0.53–1.20)	1.14 (0.74–1.74)	0.562
	Merchant	121 (51.0%)	117 (49.0%)	0.72 (0.52–1.01)	1.27 (0.90–1.79)	0.172
	Student	18 (35.3%)	33 (64.7%)	0.38 (0.21–0.70)	2.64 (1.40–4.95)	0.003*
	Daily laborer	29 (54.7%)	24 (45.3%)	0.82 (0.45–1.46)	1.19 (0.65–2.19)	0.569
Maternal history of circumcision	Yes	425 (55.3%)	343 (44.7%)	1	1	
	No	18 (34.0%)	35 (66.0%)	0.42 (0.23–0.75)	2.58 (1.41–4.71)	0.002*
Maternal Knowledge	Poor	229 (59.6%)	155 (40.4%)	1	1	
	Good	214 (49.0%)	223 (51.0%)	0.65 (0.49–0.86)	1.48 (1.12–1.98)	0.007*

* Statistically significant variables.

1, reference category

In the multivariable analysis, maternal occupation, partners’ occupation, maternal knowledge of FGM/C, and maternal history of circumcision were shown to have a statistically significant association with women’s attitudes toward FGM/C.

Accordingly, participants who were private workers in their occupation were 4 (AOR = 3.99; 95% CI: 1.63–6.77) times more likely to have unfavorable attitudes toward FGM/C than housewives. In addition, the governmental employee participants were about twofold (AOR = 2.12; 95% CI: 1.45–3.11) more likely to have unfavorable attitudes about FGM/C than housewives. Participants with a student partner were 2.6 (AOR = 2.64; 95% CI: 1.40–4.95) times more likely to have unfavorable attitudes toward FGM/C than those whose partners’ occupation was farming. The qualitative findings also supported this association. For instance, a KI said that:

“…most women who are housewives in occupation strongly promote the practice considering social relations and a problem of need (being a local celebrity); however, mothers like governmental and non-governmental workers in occupation disagree to perform the practices. But the majorities refuse now; I think they have information regarding that…” (KI2).

Participants who did not have a history of circumcision were 2.58 (AOR = 2.58; 95% CI: 1.41–4.71) times more likely to have unfavorable attitudes toward FGM than those participants who had a history of circumcision. The qualitative findings support this result. For instance, a KI was mentioned:

“…Female circumcision is our cultural practice in society, and I agree with our grandparents who decided to circumcise my daughters because the practice has different reasons like cleanliness, avoiding shame, celebration, and also cutting parts of the uterus that will grow that may harm and prevent the easy passage of the fetus during labor.” (KI51)

Also, another KI (circumciser) shared her idea and pointed out her view:

“…She may choose not to get married because she hasn't undergone circumcision, as doing so would help her gain respect and honor from the community, unless the community places no value on her family in that case. I started female circumcision years ago with the hopes that it would provide me with a source of income…” (KI101)

The likelihood of having unfavorable attitudes toward FGM/C was 1.48 times higher among participants with good knowledge of FGM/C than those with poor knowledge (AOR = 1.48; 95% CI: 1.11–1.98). The qualitative findings also supported this association. For instance, a KI said that:

“…Nowadays, the majority of society is educated and aware that it (FGM/C) is performed by untrained women with unclean blades, the bad effects of the practice on girls’ health. However, those who are uneducated and not informed about the ill effects of the practice think that the clitoris is not an important part of hygiene that causes bad odor. Also, they see the wound as a little obstacle, a wound in the Wolaita language ‘xube masuntta’…” (KI3).

### Socio-cultural attributes of FGM/C

The study explored the fact that the FGM/C practice is a long-standing tradition that has been inherited and passed down through generations. Almost all interviewees mentioned that the local culture, either in the district or the Wolaita zone, is an important influence on the existence of the FGM/C practice. The interviewees stated that the FGM/C practice is part of their culture; they want to hand it over to future generations. The socio-cultural influencers, including marriages, norms or values, family honor, shame or stigma, income sources, respect for others, a poor relationship with her husband, a decrease in external beauty, the requirement for marriage, upholding family honor, and providing local traditional circumcisers with a source of income, were explored in this study. For instance, a KI mentioned:

“…I know FGM is our gift from our parents; they instructed us to do so for different purposes, like to prevent the bad smell of girls, to have respect, to avoid shame, to celebrate, and to improve society's coherence.” (KI4)

The interviewees also mentioned that most of the families are forced to do FGM/C due to fear of stigma, to have a good relationship with their husband, thereby preventing the risk of divorce, and to increase external beauty, which will in turn attract the husband to have sexual intercourse.

Another KI added:

“…But our community perceives that the uncircumcised girls at adolescent or fire ages will break utensils at home; they will have increased sexual desire and will not be permissible to their partners; they will not be humble; they will lack cleanliness during menstruation; they will have increased vaginal secretions; their clitoris parts will grow more; and this can decrease the external beauty and affect attraction by her husband during sexual intercourse.” (KI11)

## Discussion

Since there was a higher prevalence of FGM/C practice in the study area, we hypothesized that women have poor knowledge and unfavorable attitudes toward FGM/C. Hence, this study aimed to assess women's knowledge and attitudes toward FGM/C and associated factors in the Duguna Fango district of southern Ethiopia. Accordingly, about 53.2% (CI: 49.8%, 56.5%) of women had good knowledge, and 46% (42.6%, 49.3%) had unfavorable attitudes (against FGM/C). Monthly income and partners’ education status were identified as factors affecting women's knowledge of FGM/C. Moreover, maternal occupation, partners’ occupation, maternal knowledge of FGM/C, and maternal history of circumcision were found to be factors influencing women's attitudes toward FGM/C.

The proportion of good knowledge found in this study is lower compared to proportions from Kenya (85.4%) (14), Sudan (85%) (24), and Jigjiga (88.3%) (25). This variation can be explained by the availability and implementation (enforcement) of different anti-FGM/C laws. Also, it might be due to the inconsistency in the study tools utilized. For instance, studies on Kenya and Sudan have focused more on the health consequences of FGM/C. Moreover, the promising justification for the higher proportion of good knowledge in Jigjiga City might be associated with different (governmental and non-governmental) agents’ unwavering attention given to increasing public awareness and ending the highest FGM/C practice in the region (26). The current finding is consistent with the studies conducted in the Degadamot district of Amhara Regional State and Kembata Tambaro zones, where 56.6% and 55.4% of women had good knowledge of FGM/C, respectively (21, 27). However, the current finding is higher than the finding from the Hababo Guduru District, where 46.1% of women had good knowledge (26). A possible explanation for this variation could be the difference in the studies’ time and sample size, where the current study utilized a larger sample size.

The unfavorable attitudes toward FGM/C found in the current study are lower than the local and global findings, including the studies from Nigeria (70%), Sudan (67%), Jigjiga district (62.7%), and Hababo Guduru District, Western Ethiopia (53%) (20, 24–26). The difference found between the current and previous findings (Nigeria and Sudan) might be related to differences in socio-demographic features across countries, the presence or absence of FGM/C laws, and health system responses. In addition, the discrepancy observed between the current finding and local (the Jigjiga district and Hababo Guduru district) findings could be linked to socio-demographic variations.

The current study identified that the participants’ monthly income and partners’ educational status were significantly associated with the women's knowledge of FGM/C. Accordingly, the participants who earn about 500–999 ETB per month were about 61% more likely to have a good knowledge of FGM than those who earn less than 500 ETB. This finding is congruent with a finding from the Degadamot district of Amhara Regional State (21). This finding supports the fact that women who earn a higher income may have more access to information related to FGM/C.

The educational status of the partner was found to be significantly associated with the woman's knowledge of FGM/C. Accordingly, participants whose partners attended college and above were about two times more likely to have a good knowledge of FGM/C compared to participants’ partners who did not attend formal education. A study conducted by Belda SS. et al. supported this finding (38). This finding is plausible because the more educated the partner is, the more he is exposed to health information, and the more his wife benefits.

In the current study, the participants who worked in the private sector were four times more likely to have unfavorable attitudes toward FGM/C compared to those who were housewives. Also, government employees were twofold more likely to have unfavorable attitudes toward FGM/C than those who were housewives. This finding is in line with a finding from Egypt (30). Private sector and government employees may be aware of FGM/C as a crime that can result in punishment through training or exposure to different media.

In this study, participants who had a student partner were 2.6 times more likely to have unfavorable attitudes toward FGM/C compared to those whose partners’ occupation was farming. This association was not supported by previous literature. However, it might be due to student partners learning the harmful consequences of FGM/C from various sources and being able to share this information with the mother, which could lead to favorable attitudes toward FGM/C.

Moreover, the participants who had no history of circumcision were more than two times more likely to have a unfavorable attitude toward FGM/C compared to those who were circumcised. This finding is consistent with a study conducted in Egypt (29). Again, this finding was supported by the qualitative finding. Accordingly, the circumcised mothers supported FGM/C because they believed in the tradition or because of the family and social pressure in the community.

The likelihood of having unfavorable attitudes toward FGM/C was about 1.48 times higher among participants who had good knowledge of FGM/C compared to their counterparts. This finding is consistent with the studies from southern Nigeria and Egypt (23, 29). This is also a plausible finding related to natural truth. An individual with good knowledge of a certain health phenomenon has a favorable attitude toward that phenomenon.

In addition, the qualitative study explored the sociocultural influencers and poor awareness about the adverse effects of FGM/C as influences on women's knowledge and attitudes. This was also explored in a qualitative study conducted in the United Kingdom (36). Controlling sexuality and wrong religious prescriptions were also identified as factors influencing women's knowledge and attitudes toward FGM/C. This is supported by a finding from the USA that explored motivation for social acceptance, marriageability, or proof of virginity, reducing promiscuity, and religion as contributors to FGM/C (13). Contrary to this, a study from Oslo discovered that no godly laws support FGM/C and that it is a traditional practice (33). This might be due to the variations in the religious and cultural perceptions in the two countries.

### Strength and limitations

This study utilized qualitative and quantitative data at the community level to identify and explore the socio-cultural and religious influencers of FGM/C knowledge and attitudes. Moreover, the qualitative part of the study was conducted and reported according to the three domains of the consolidated criteria for reporting qualitative research (COREQ) checklist (Supplementary S3). However, because of its cross-sectional nature, it could not assure cause-and-effect relationships. Moreover, the model complexity should be considered while interpreting the findings.

## Conclusion

This mixed approach study found poor insights of women toward FGM/C. Sociodemographic factors, including educational status and occupation, were identified as influencers of women's attitudes toward FGM/C. In addition, this study explored sociocultural perceptions, awareness of FGM/C, religious implications, and sexuality as attributes of women's knowledge and attitudes toward FGM/C. Hence, these findings call for community interventions targeted to increase insights toward FGM/C.

## Data Availability

The original contributions presented in the study are included in the article/Supplementary Material, further inquiries can be directed to the corresponding author.

## References

[1] WHO. Management of Health Complications from Female Genital Mutilation WHO Guidelines on the (2018). 27359024

[2] World Health Organisation. Female Genital Mutilation. Fact Sheet (2012), Vol. 241.

[3] United Nations Children’s Fund. Female Genital Mutilation/Cutting: What might the future hold? New York: UNICEF (2014).

[4] World Health Organization. Female Genital Mutilation Hurts Women and Economies. Geneva: World Health Organization (2020). Available at: https://www.who.int/news/item/06-02-2020-female-genital-mutilation-hurts-women-and-economies#:∼:text=Treating%20female%20genital%20mutilation%20costs%20USD%201.4%20billion%20per%20year%20globally:%20WHO&text=Female%20genital%20mutilation%20(FGM)%20exacts,World%20Health%20Organization%20(WHO) (Accessed April 7, 2025).

[5] Tordrup D Bishop C Green N Petzold M Vallejo FR Vogel JP Economic burden of female genital mutilation in 27 high-prevalence countries. BMJ Glob Health. (2022) 7(2):e004512. 10.1136/bmjgh-2020-004512 35105556 PMC8744099

[6] Moges T. Knowledge, attitude and practice of female genital mutilation among the community of Gursum Woreda, Somali regional state of eastern Ethiopia (diss. MA thesis). Addis Ababa University (2017).

[7] India VS. IFMSA Policy Proposal Harmful Traditional Practices. Denmark: IFMSA (2022). Available at: https://scholar.google.com/scholar?hl=en&as_sdt=0%2C5&q=+IFMSA+Policy+Proposal+Harmful+Traditional+Practices+%282022%29&btnG=#d=gs_cit&t=1743921015436&u=%2Fscholar%3Fq%3Dinfo%3ABpDyvor77CYJ%3Ascholar.google.com%2F%26output%3Dcite%26scirp%3D0%26hl%3Den (Accessed April 7, 2025).

[8] UNICEF for every child. Female Genital Mutilation in the Middle East and North Africa. Available online at: https://www.unicef.org (Accessed June 23, 2024).

[9] UNICEF. Female Genital Mutilation/Cutting in Ethiopia. Luxembourg: UNICEF (2021). Available at: https://scholar.google.com/scholar?hl=en&as_sdt=0%2C5&q=Female+Genital+Mutilation%2FCutting+in+Ethiopia+-+EUAA&btnG=#d=gs_cit&t=1743930488152&u=%2Fscholar%3Fq%3Dinfo%3AxcwpmXFJFncJ%3Ascholar.google.com%2F%26output%3Dcite%26scirp%3D0%26hl%3Den (Accessed April 7, 2025).

[10] Central Statistical Agency (CSA) [Ethiopia] and ICF. Ethiopia Demographic and Health Survey 2016. Addis Ababa, Ethiopia: CSA/ Rockville, MD: ICF (2016).

[11] Awolola OO Ilupeju NA. Female genital mutilation; culture, religion, and medicalization, where do we direct our searchlights for its eradication: Nigeria as a case study. Tzu Chi Med J. (2019) 31(1):1–4. 30692824 10.4103/tcmj.tcmj_127_18 PMC6334568

[12] Wander K Shell-Duncan B. Social norm coordination and readiness to change female genital cutting: evidence from Senegambia. SSM Popul Health. (2020) 11:100593. 10.1016/j.ssmph.2020.100593 32490136 PMC7256638

[13] Avolio BJ Waldman DA Einstein WO. Organizational ethics in a management game simulation. Group Organ Stud. (1988) 13(1):59–80. Understanding the association between parental attitudes and the practice of female genital mutilation among daughters. (2020):1–10. 10.1177/105960118801300109

[14] Jaldesa G Guyo A Askew I Njue C Wanjiru M. Female genital cutting among the Somali of Kenya and management of its complications. In FRONTIERS Final Report. Washington, DC: Population Council (2005).

[15] Lien IL Schultz JH. Internalizing knowledge and changing attitudes to female genital cutting/mutilation. Obstet Gynecol Int. (2013) 2013:467028. 10.1155/2013/467028 23843795 PMC3694526

[16] Greenberg PE Fournier A-A Sisitsky T Pike CT Kessler RC. The economic burden of adults with major depressive disorder in the United States (2005 and 2010). J Clin Psychiatry. (2015) 76(2):155–62. 10.4088/JCP.14m09298 25742202

[17] Malak M Basalem D Aleiidi S Helabi N Almutairi M Alhamed A. Awareness of female genital mutilation/cutting among the general population in 2019: a survey-based study in Saudi Arabia. Cureus. (2020) 12(1):e6651. 10.7759/cureus.6651 31949997 PMC6959839

[18] Africa WHO. Ethiopia Bans Medicalization of Female Genital Mutilation (FGM). Geneva: Africa WHO (2017). Available at: https://scholar.google.com/scholar?hl=en&as_sdt=0%2C5&q=WHO.+Ethiopia+Bans+Medicalization+of+Female+Genital+Mutilation+%28FGM%29.WHO++%282017%29.&btnG= (Accessed April 7, 2025).

[19] Ahmed HM Shabu SA Shabila NP. A qualitative assessment of women’s perspectives and experience of female genital mutilation in Iraqi Kurdistan Region. BMC Womens Health. (2019) 19(1):1–12. 10.1186/s12905-019-0765-7 31096978 PMC6521410

[20] Victoria AF Victoria OT Kayode NH Monsurat N. Knowledge and attitude of female genital mutilation among mothers living in afao community. Ekiti State. (2022) 2(4):116–23. Available at: https://www.dzarc.com/education/article/view/231

[21] Melese G Tesfa M Sharew Y Mehare T. Knowledge, attitude, practice, and predictors of female genital mutilation in Degadamot district, Amhara regional state, Northwest Ethiopia, 2018. BMC Womens Health. (2020) 20(1):1–9. 10.1186/s12905-020-01041-2 32795298 PMC7427771

[22] Abathun AD Gele AA Sundby J. Attitude towards the practice of female genital cutting among school boys and girls in Somali and Harari Regions, Eastern Ethiopia. Obstet Gynecol Int. (2017) 2017:1–9. 10.1155/2017/1567368 PMC5366198 28386281

[23] Obi AI Obarisiagbon OE Igbinadolor OL Fatai KM Adesoye OO. Factors associated with the knowledge and attitude towards female genital mutilation among antenatal clinic attendees in Southern Nigeria. Health Res. (2019) 5(2):183–92. 10.30442/ahr.0502-20-50

[24] Esmeal EA Mohammed Ahmed AE Waggiallah HA Almosaad YM. Knowledge, attitude, and practice among mothers towards female circumcision Ombada province Khartoum state, Sudan. Int J Community Med Public Health. (2016) 3(7):1788–94. 10.18203/2394-6040.ijcmph20162043

[25] Gebremariam K Assefa D Weldegebreal F. Prevalence and associated factors of female genital cutting among young adult females in Jigjiga district, eastern Ethiopia: a cross-sectional mixed study. Int J Womens Health. (2016) 8:357–65. 10.2147/IJWH.S11109 27563257 PMC4984990

[26] Gajaa M Wakgari N Kebede Y Derseh L. Prevalence and associated factors of circumcision among daughters of reproductive aged women in the Hababo Guduru District, Western Ethiopia: a cross-sectional study. BMC Womens Health. (2016) 16(1):1–9. 10.1186/s12905-016-0322-6 27449648 PMC4957895

[27] Awol A. Knowledge, Attitude and Practice (KAP) of Women Towards Female Genital Mutilation, Angacha Kembataa. Munich: GRIN Verlag (2017). Available online at: https://www.grin.com/document/423650 angacha kembataa.

[28] Abolfotouh SM Ebrahim AZ Abolfotouh MA. Awareness and predictors of female genital mutilation/cutting among young health advocates. Int J Womens Health. (2015) 7:259–69. 10.2147/IJWH.S78664 25759602 PMC4346006

[29] Abdou MS Wahdan IM El-Nimr NA. Prevalence of female genital mutilation, and women’s knowledge, attitude, and intention to practice in Egypt: a nationwide survey. J High Inst Public Health. (2020) 50(3):139–45. 10.21608/jhiph.2020.121424

[30] Van Rossem R Meekers D Gage AJ. Women's position and attitudes towards female genital mutilation in Egypt: a secondary analysis of the Egypt demographic and health surveys, 1995–2014. BMC Public Health. (2015) 15:874. 10.1186/s12889-015-2203-6 26357927 PMC4566495

[31] Pankhurst A. Child Marriage and Female Circumcision (FGM/C): Evidence from Ethiopia. Young Lives (2014). Available at: https://ora.ox.ac.uk/objects/uuid:70808644-dee9-408c-9162-de0ec3f317b8/files/m7e11c2c88524ed533144715fa7a96702 (Accessed April 9, 2025).

[32] Anjulo BB Lambebo AF. Prevalence and associated factors of female genital mutilation among reproductive age women’s of Wolayita Zone, Southern Ethiopia: A cross-sectional study. Int J Sex Reprod Health Care. (2021) 4(1):091–8. 10.17352/ijsrhc.000030

[33] Gele AA Kumar B Hjelde KH Sundby J. Attitudes toward female circumcision among Somali immigrants in Oslo: a qualitative study. Int J Womens Health. (2012) 4(1):7–17. 10.2147/IJWH.S27577 22312195 PMC3271810

[34] Tong A Sainsbury P Craig J. Consolidated criteria for reporting qualitative research (COREQ): a 32-item checklist for interviews and focus groups. Int J Qual Health Care. (2007) 19(6):349–57. 10.1093/intqhc/mzm042 17872937

[35] Declaration of Helsinki. Recommendations guiding doctors in clinical research. Adopted by the World Medical Association in 1964. Wis Med J. (1967) 66(1):25–6. 5340792

[36] Ali S De Viggiani N Abzhaparova A Salmon D Gray S. Exploring young people’s interpretations of female genital mutilation in the UK using a community-based participatory research approach. BMC Public Health. (2020) 20(1):1–15. 10.1186/s12889-019-7969-5 32689963 PMC7370427

[37] Yoder PS Wang S. Female Genital Cutting: The Interpretation of Recent DHS Data. DHS Comparative Reports No 33. Calverton, Maryland, USA: ICF International (2013).

[38] Belda SS Tololu AK. Knowledge, attitude and practice of mothers towards female genital mutilation in South West Shoa zone, Oromia region, Ethiopia. MOJ Public Health. (2017) 6(2):279–86. 10.15406/mojph.2017.06.00162

