# Supplementary material for: Women's knowledge and attitudes toward female genital mutilation and associated factors in Diguna Fango, a rural district in southern Ethiopia: a community-based mixed study
Source: Front Glob Womens Health. 2025 Apr 15;6:1516925. doi: 10.3389/fgwh.2025.1516925 (PMC12037511; doi:10.3389/fgwh.2025.1516925)
Supplement: Supplementary file 1 [file Table1.docx]

English version questionnaire

Section I- Socio-demographic characteristics

| S/N | Questioners | Responsible answer | Skip |
| --- | --- | --- | --- |
| 1 | How old are you? | age in year __________________ |  |
| 2 | What is your religion? | 1.Orthodox  2.Protestant  3.Muslim  4.Catholic  5.Others (specify______) |  |
| 3 | What is your ethnicity? | 1.Wolaytta  2.Dawuro  3.Gamoo  4.Others(specify)________ |  |
| 4 | What is your residence? | 1. urban  2. rural |  |
| 5 | What is your educational status? | 1. No formal education  2.Primary (1-8 grades)  3.Secondary (9-12grades)  4.Above secondary (above grades) |  |
| 6 | What is your occupation? | 1. House wife 2. Civil servants 3. Gov’tal employment 4. Merchant 5. Student 6. Others 7. (specify)______________ |  |
| 7 | What is your marital status? | 1. Single  2. Married  3. Divorced  4. Widowed  5. Separated |  |
| 8 | What is your partners’ education status? | 1. No formal education 2. Primary 3. Secondary 4. Above secondary |  |
| 9 | What is your partner’s occupation? | 1. Farmer 2. Gov’tal/ NGO employment 3. Merchant 4. Student 5. Daily workers 6. Others (specify)_____________ |  |
| 10 | What is your family monthly income? | _______________in Eth. Birr |  |

Section II- The questions related to knowledge towards FGM

| S/N | Questionnaires | Responses | Skip |
| --- | --- | --- | --- |
| 11 | Do you ever awared about female genital circumcision? | 1.Yes  0. No |  |
| 12 | Do you know FGM has health complication? | 1.Yes  0. No |  |
| 13 | Could you classify it as immediate or long complication | 1.Yes  0. No |  |
| 14 | Do you know FGM can decrease sexual pleasure? | 1.Yes  0. No |  |
| 15 | Do you know FGM is harmful traditional practices? | 1.Yes  0. No |  |
| 16 | Do you know FGM brings complication during delivery? | 1.Yes  0. No |  |
| 17 | Do you know the FGM has no health benefits? | 1.Yes  0. No |  |
| 18 | Do you know FGM is forbidden in the law? | 1.Yes  0. No |  |
| 19 | Do you know FGM is a violation of the rights of the girl-child | 1.Yes  0. No |  |
| 20 | Do you know the different forms of FGC? | 1.Yes  0. No |  |

Section III- Questions related to parental factors towards female genital cutting

| S/N | Questionnaire s | Responses | Skip |
| --- | --- | --- | --- |
| 21 | Who decides to perform FGM? | 1. Father 2. Mother 3. Both mother and father 4. Grand Parents 5. The girl herself 6. Relatives 7. Others (specify _____ 8. I do not know |  |
| 22 | Do you have history of ANC visit / place of delivery at health institution? | 1. Yes 2. No |  |
| 23 | Do you have circumcised? | 1. Yes 2. No ` 3. I do not know |  |
| 24 | Do your family support for the continuation of the FGC. | 1. Yes 2. No 3. I do not know |  |
| 25 | Do know the reason of your family support of the continuation of the FGC. | 1. Yes 2. No 3. I do not know |  |
| 26 | What do you think is the best way to stop female circumcision? | Multiple answers are possible!   1. Enforced legislation 2. Educational campaign to women 3. Improvement of status of women 4. Fathers should take more responsibility 5. Sexual education 6. Others (Specify)   99. No Response |  |

Section III- Questions related to attitude towards female genital cutting

| S/N | Questionnaires | Responses | Skip |
| --- | --- | --- | --- |
| 27 | Do you support FGM? | 1.Strongly disagree  2.Disagree  3.Neutral  4.Agree  5.Strongly agree |  |
| 28 | Does FGM can protect virginity of female? | 1.Strongly disagree  2.Disagree  3.Neutral  4.Agree  5.Strongly agree |  |
| 39 | Do you think uncircumcised females are not faithful for marriages? | 1.Strongly disagree  2.Disagree  3.Neutral  4.Agree  5.Strongly agree |  |
| 30 | Do you think that this practice is required by your religion? | 1.Strongly disagree  2.Disagree  3.Neutral  4.Agree  5.Strongly agree |  |
| 31 | Do you think uncircumcised females have increased sexual feeling? | 1.Strongly disagree  2.Disagree  3.Neutral  4.Agree  5.Strongly agree |  |
| 32 | Do you think FGM is good practice? | 1.Strongly disagree  2.Disagree  3.Neutral  4.Agree  5.Strongly agree |  |
| 33 | Do you think uncircumcised female has problem during child birth? | 1.Strongly disagree  2.Disagree  3.Neutral  4.Agree  5.Strongly agree |  |
| 34 | Will you voluntarily circumcise if you have daughter? | 1.Strongly disagree  2.Disagree  3.Neutral  4.Agree  5.Strongly agree |  |
| 35 | Do you think uncircumcised female calls as a maid in societies? | 1.Strongly disagree  2.Disagree  3.Neutral  4.Agree  5.Strongly agree |  |
| 36 | Do you agree with FGM continuity for the future? | 1.Strongly disagree  2.Disagree  3.Neutral  4.Agree  5.Strongly agree |  |
