# Supplementary material for: Women's knowledge and attitudes toward female genital mutilation and associated factors in Diguna Fango, a rural district in southern Ethiopia: a community-based mixed study
Source: Front Glob Womens Health. 2025 Apr 15;6:1516925. doi: 10.3389/fgwh.2025.1516925 (PMC12037511; doi:10.3389/fgwh.2025.1516925)
Supplement: Supplementary file 2 [file Table2.docx]

**Annex VI: Information Sheet (Wolaitatto version)**

**Wolaytatto doonaa naqaasha odiyaa kifiliyaa**

Sarotaa

Ta Sunttay_______________________________

Ha Pilggettaa halchchoy Tohossa Toophphiyaan, Wolaita Ambban, Duguna panggo allanaa garssan macca naatu qaxaraban layttay (15-49) giddon de’’iyaa aayetu eraa, xeelanne haraa gayto qofataa akekanassa.Intte ha pilggettaa oosuwawu kushshetanawu doorettideta. Ha pilggetta nababboy pollettiyo hanottati oyshshaa qommuwan.Gidoppe attin nu gidoni ha Pilggettaraa oykketida yohuwaan haga taani koyikke gidi qaalla laammiyogaa diggiyay baynaggatto woykko maatay deiyogatto qoncissaysi. Leemisuwasi nababbuwaara woykko inttena inte de’’uwan gakkidabay deikko un’’ettenagatto odayssi. Taani ha pilggetta kumetta hanottan holliyawu gattanawu inttena ammanettidanee sittanne tumu qofaa ha shiqqida oyshatussi zaaro demmiyoge keehippe maddiyagane qassi xoqqa hanottan inttena sabbisiyagaa. Haga tanara oottanawu intte sheniyaa taayo immiyogappe attin inttena marccuwaa maqaciyay baynnaga gidiyogatto qonccissaysi. Harabay qassi tammanne hosppun layttappe garssa gidiyaa aayetu gishshaa inttena yelageeti woykko intte keettaway oyshetes .

Hode intte ha kaallidi deiyaa oychchana qofatusi kushshaa akkekiko nuuni sinttawu baanawu danddayettes.

**Ha Pilggettaa hollee:** Tohossa Toophphiyaan, Wolaita Ambban duguna panggo allanaa garssan maccaa naatu qaxaraban layttay (15-49) giddon de’’iyaa aayetu eraa, xeelanne haraa gayto qofataa akekanassa..

**Madduwaa:** Intte ha pilggetta oosuwawu kushshetiyogan aybane marccuwaa maaddoynne kaafe baynnagatto erissays.

**Qohiyaba**: Intte taanara ha pilggetaa oosuwawu kushshetiyogan qohiyabay gakkana danddayes giidi qoppopite.

**Xuura xeelliyagan** :Taani intteyo qonccissiyobay deikko ha naqashsha xuuray ha pilggetta nababbo xalalasa gidiyooga erissaysi. Hegaa gishshawu oyshsha taani oychchiyode intte immiyo naqashshay xuuran geemmidi ha pilggettta xalalan go’’ettiyogaa gidishin intte oonatettay woyko sunttay aybanne gelenaga giddiyoga erissays.

**Koshshiyabawu gayttiyo gadawaa:**Ha pilggetta oosuwaa oottiya gadawaa oychchanawu koyyido wodee deikko ha garssara immettida silkee payduwaan woykko emaile carkuwaa yafaran shociyogan woykko kitaa xaafiyoogan demmanawu danddeyeta: Silkee Paydoy: +251900500364 woykko E-maile: beyenetame@gmail.com giyaganaa.

**Annex VII: consent/assent form (Wolaitato version)**

Wolaytatto doonan ishshatettane eenotetta qonccissuwaa odiyaa kifiliyaa

Taani intteyo qoncissiyobay deikko ha naqashshayi xuuratettan ha pilggetta ooso xalalasa gidiyoga erissaysi. Hega gishshawu oyshaa taani oychiyode intte immiyo naqashshay xuuran geemettidi ha pilggettta xalalan go’’ettiyoga gidishshin intte oonatettay woyko sunttay aybanne gelennaga giddiyoga erissays. Issi Issiba inttiyo qonccissogappe guyiyan intekka akkekisuwaa deemmidi ishshi giikko qassi hagan ha pilggetta oosuwawu kushetanawu mayettikko doommana dandayoos.Qassi qofana bessiyage’taani taappe koyyettiyabay aybako akkekadanne qassi taani ha oosuwaan/pilggettan tana xeelliyaga oottin aybi hananakko erasi. Taana Loida wodiyan pilggetta oosuwaa xeelliyagan taana hassanawu danddayiyogaka akekkasi.

Neeyo ayba oyshi deaneeshsha? Yaatin ha Oyshaa zaaranawu mayettideti?

Maayettike__________(Galataysi ne essa) Maayettassi______________(Galatays ne doomma)

Oysha kuriya ____________________

Naqasha shishshiyaga Sunttaa___________________ paramuwaa___________Gaal_________

Kaallidi xeelliyagaa Suntta_____________________Paramuwaa__________ Gaal____

Koyro Shempuwa: Zaaruwaa immiya aayetu heeranne deretettaa Naqqashaa

| Maaraa payduwa | Oyshata | Bessiyaa zaarota | Xaaluwaa |
| --- | --- | --- | --- |
| 1 | Laytta (Bariyaa)? | __________________ |  |
| 2 | Ammanuwaa qommuwaa? | 1.Orthodoxiyaa  2.Protestantiyaa  3.Muslimiyaa  4.Catholikiyaa  5.Harrata____ |  |
| 3 | Yeletaa qommuwaa? | 1.Wolaytta  2.Dawuro  3.Gamoo  4. Harrataa ________ |  |
| 4 | Diyyo sohuwa? | 1. Katamaa (Ambbaa)  2. Gaxariyaa (Gandda) |  |
| 5 | Timirttiyaa xekkaa? | 1. Tamaaribenagaa  2. Kifile 1-8 tamaridagaa)  3. Kifile 9-12 tamaridagaa  4.12nttape bolla kifiliyaa tamaridagaa |  |
| 6 | Oosuwa qommuwaa? | 1.So giddo osaanchcha  2.Goshshaa  3.Kawo oosuwaa  4.Zal’’iyaa  5.tammarre  6.Dummatiya oosota gidikko qonccissa______________ |  |
| 7 | Machchiyo/geliyo hanotaa? | 1. Machchibennagaa  2. Machchidaagaa  3. Machchidi birshshidagaa  4. Keettawayi hayqqidooro |  |
| 8 | Keettaawaa timirttiyaa xekkaa? | 1. Tamaaribenaagaa  2. Kifile 1-8 tamaridaggaa)  3. Kifile 9-12 tamaridaggaa  4. 12nttape bolla kifiliyaa tamaridagaa |  |
| 9 | Keettaawaa Oosuwa qommuwaa? | 1.Goshshaa  2.Kawo oosuwaa  3.Zal’’iyaa  4.tamaare (Luxiyaagaa)  5Wolqqa oossancha.  6.Dummatiya oosota gidikko qonccissa______________ |  |
| 10 | So asaassi aginan geliyaa miishshaa qooday woyse? | _______________Toophphiyyaa mishsshaani |  |

Naa’’antto Shemppuwaa: Macca naatu qaxaraa xeelliyagan aayetusi de’’iyaa eraa pilggiyaa ayfe oyshata

| M/payduwaa | Oyshata | Bessiyaa zaarota | Xaaluwaa |
| --- | --- | --- | --- |
| 11 | Hagaape kase wodiyan macca naatu qaxaraba siyiddi ereeti | 1.Ee  0.Chii |  |
| 12 | Macca naatu qaxaray payyatetta bollan mettuwaa gattiyogaa ereeti? | 1.Ee  0.Chii |  |
| 13 | Ha mettota, mata woyko adussa wodiyan gakkiyaa mettota giidi shaakidi ereeti? | 1.Ee  0.Chii |  |
| 14 | Macca naatu qaxaray mattumaa gaytotettaa ufayssaa guutiyogaa ereeti ? | 1.Ee  0.Chii |  |
| 15 | Macca naatu qaxaray qohiyaa meeze woga gidiyogaa ereeti? | 1.Ee  0.Chii |  |
| 16 | Macca naatu qaxaray yelliyo wodiyan mettuwaa gattiyoga ereeti | 1.Ee  0.Chii |  |
| 17 | Macca naatu qaxarassi payyatetta bollani go’’ay de’’iyoga ereeti? | 1.Ee  0.Chii |  |
| 18 | Macca naatu qaxaray kawotettaa seeran teqettiyogaa ereeti? | 1.Ee  0.Chii |  |
| 19 | Macca naatu qaxaray guutta naatu maataa yedhdhiyoga ereeti? | 1.Ee  0.Chii |  |
| 20 | Dumma dumma macca naatu qaxaraa qommotaa ereeti ? | 1.Ee  0.Chii |  |

Heezza’’antto Shemppuwaa: Maccaa naattu qaxaaraa xeliyagan yelidagetura gayttiyaa ayfe Gidiyaa Oyshata

| M/payduwaa | Oyshataa | Bessiyya zaarotta | Xaaluwaa |
| --- | --- | --- | --- |
| 21 | Intte heeran Macca naata qaxaranassi kuushsha qofaa immiyay oone ? | 1.Aawaa  2.Aayiyoo  3. Aawaa/Aayiyo yelidageta  4.Qaxarettiya na’’iyoo  5.Dabbo asaa  6.Haraata  7.Taani erike |  |
| 22 | Hagape kase abbe ixetaa wodiyan xeelletanawu woyko yelanawu payatetta naago keetta biidi ereeti? | 1.Ee  0.Chii |  |
| 23 | Beni intte qaxarettidetiyye? | 1.Ee  0.Chii |  |
| 24 | Intte soo asay macca naati qaxaretana mala koyyiye?. | 1.Ee  0.Chii |  |
| 25 | Eshi koyyiko gaasoy aybako ereeti?. | 1.Ee  0.Chii |  |
| 26 | Macca naatu qaxaraa xayissiyo lo’’o qofay ayba giidi qopeeti? | !Issuwappe daruwaa dooranawu danddayettes  1.Kawotetta seeraa minttiyoga  2.Macca naata tamaarissiyogaa  3.Yelidagetussi daroo hadaraa immiyogaa  4.Taani erike |  |

Oydda’’antto shemppuwaa: Macca naatu qaxaraa bollan de’iyaa aayetu xeelaa oychchiya ayfe gidiyaa oyshata

| M/payduwaa | Oyshata | Bessiyaa zaarota | Xaaluwaa | | |
| --- | --- | --- | --- | --- | --- |
| 27 | Macca naatu qaxaray de’’iyoga maayettay neeni? | 1.Kehippe phalqqays  2. phalqqays  3.taani erikee  4.maayettays  5. Kehippe maayettays | |  | |
| 28 | Macca naatu qaxaray geelo’’ttetan de’’iddi mattuma gaytotteta polenadan naagees? | 1.Kehippe phalqqays  2. phalqqays  3.taani erikee  4.maayettays  5. Kehippe maayettays | |  | |
| 29 | Qaxaretibena macca naati keetta awaraa de’’iyo duusawu ammantokona giidi qopettiye ? | 1.Kehippe phalqqays  2. phalqqays  3.taani erikee  4.maayettays  5. Kehippe maayettays | |  | |
| 30 | Macca naatu qaxaraa intte ammanoy koyyes giidi qopettiye? | 1.Kehippe phalqqays  2. phalqqays  3.taani erikee  4.maayettays  5. Kehippe maayettays | |  | |
| 31 | Qaxaretibena macca naati mattuma gaytotteta qofay guuta giidi qopettiye? | 1.Kehippe phalqqays  2. phalqqays  3.taani erikee  4.maayettays  5. Kehippe maayettays | |  | |
| 32 | Macca naatu qaxaray lo’’o meeze giidi qopettiye? | 1.Kehippe phalqqays  2. phalqqays  3.taani erikee  4.maayettays  5.Kehippe maayettays. | |  | |
| 33 | Qaxaretibena macca naatussi yeliyo wodiyn mettoy de’’es giidi qopettiye? | 1. Kehippe phalqqays 2. phalqqays 3. taani erikee 4. .maayettays 5. Kehippe maayettays | |  | |
| 34 | Sinttanawu neeni yeliddo na’’iyaa qaxaretanawu mayettay? | 1.Kehippe phalqqays  2. phalqqays  3.taani erikee  4.maayettays  5.Kehippe maayettays | |  | |
| 35 | Intte heerato qaxaretibena macca naati soo garssa oosanchchageetetta xeesetawusu gaada qoppay? | 1.Kehippe phalqqays  2. phalqqays  3.taani erikee  4.maayettays  5.Kehippe maayettays | |  | |
|  | | | | |  |
| 36 | Macca naatu qaxaray wogay sinttawuka de’’iyogaa neeni mayettay? | 1.Kehippe phalqqays  2. phalqqays  3.taani erikee  4.maayettays  5.Kehippe maayettays | |  | |
